# Childhood Experiences of Alternative Care and Callousness/Unemotionality: A Conceptual Model, Scoping Review, and Research Agenda

**DOI:** 10.1007/s10567-023-00445-4

**Published:** 2023-07-12

**Authors:** Dave S. Pasalich, Benjamin Aquilina, Alison Hassall, Natalie Goulter, Nakiya Xyrakis, Anderson Khoo

**Affiliations:** 1 School of Medicine and Psychology, Australian National University, Building 39, Science Rd, ACT 2601, Canberra, Australia; 2 School of Psychology, Newcastle University, Newcastle upon Tyne, UK

**Keywords:** Foster care; out-of-home care; callous-unemotional traits; psychopathy; attachment; trauma

## Abstract

Individuals with experiences of alternative care (AC; i.e., out-of-home care and institutional care) are at high risk for various mental health and relational problems stemming from exposure to serious attachment disruptions, loss, and complex trauma. Yet, despite the interpersonal context of their significant adversities, surprisingly there is scant research explicitly focusing on callousness/unemotionality (e.g., lack of guilt, callous disregard for others) in this population. This paper provides the first conceptual model for, and systematic scoping review of, callousness/unemotionality in children and young people with experiences of AC. In a comprehensive search of nine databases, 22 articles involving samples of participants in AC or with histories of AC, were identified for inclusion. The pattern of findings revealed that callous-unemotional and psychopathic traits are elevated in children and young people with AC experiences, and positively associated with AC. Moreover, results showed associations between these traits and various psychosocial correlates, most consistently with externalizing and internalizing problems and attachment-related problems. Only two intervention studies were located, one of which found benefits of training and supporting foster caregivers for reducing callous-unemotional traits. These findings are discussed with respect to gaps in the literature, future research directions, and trauma-informed practice to assess and treat callousness/unemotionality in children and young people with experiences of AC.

**Supplementary Information:**

The online version contains supplementary material available at 10.1007/s10567-023-00445-4.

Children experience interpersonal trauma and risk for ongoing attachment disruptions when they are separated from or lose their primary caregiver after been orphaned, abandoned at an early age because of poverty or other socioeconomic factors, or removed from birth parents due to child protection concerns (Humphreys, 2019). When children lose a parent or are unable to live with them, they are placed in one of several different alternative care (AC) arrangements. This paper focuses on formal AC (i.e., state or court placement) which includes relative/kinship care, foster care, residential care (provision of care in small group settings with high caregiver-to-child ratios), and institutional care^1^ (provision of care in large group settings with low caregiver-to-child ratios). Most Western countries have joined universal calls for deinstitutionalization and only provide out-of-home care (i.e., kinship care, foster care, and residential care); however, institutional care is still common in some non-Western countries (Roche, 2019).

Global estimates suggest there may be upwards of 2.7 million children and young people living in formal AC (Petrowski et al., 2017). The most important determinant of positive child development and wellbeing in AC is the quality and consistency of caregiving (Harden, 2004). Children exposed to even the most serious neglect and caregiver deprivation early in life, such as orphaned children who were abandoned to institutions in Romania, can show recovery in mental health if they receive stable and nurturing caregiving in a safe home environment in AC (Wade et al., 2022).

Although some children raised in AC show remarkable resilience, there is a large literature linking experiences of AC to adverse developmental and mental health outcomes. For instance, evidence from systematic and meta-analytic reviews of mental health problems in out-of-home care suggests that compared to their peers raised in birth families, children in foster and kinship care experience more externalizing, internalizing, and attachment problems (Dubois-Comtois et al., 2021; Engler et al., 2022). Moreover, a recent review of the literature on children raised in institutional care reported significant cognitive, behavioral, attachment, and developmental issues (Gunnar & Reid, 2019).

These prior research reviews offer important understanding into problematic mental health and relational outcomes in individuals with experiences of AC. However, despite the significant interpersonal context of their early life adversities, past reviews have not reported on the potential link between AC experiences and callousness/unemotionality. This research gap is very surprising because a callous-unemotional interpersonal style is associated with a particular profile of risk factors and outcomes which is also linked to AC; namely, externalizing problems and other psychopathology, interpersonal trauma, and attachment difficulties.

## Callous-Unemotional/Psychopathic Traits

Callous-unemotional (CU) traits include reduced guilt, remorse, and empathy, and limited concern for others (Frick, 2004). In childhood, these traits significantly increase risk for externalizing problems, particularly aggression and delinquency, and predict later antisocial behaviors in adulthood (McMahon et al., 2010). CU traits capture the affective dimension of psychopathy; the other two dimensions of psychopathic traits include behavioral (e.g., reckless and impulsive behavior) and interpersonal (e.g., narcissism and deceitfulness) features. Accordingly, CU traits are studied exclusively or in the context of the broader model of psychopathic traits, and inform approaches to conceptualizing and reducing serious and persistent antisocial behavior across the lifespan (Frick & Ray, 2015). It is also important to clarify that CU traits observed in childhood are malleable and are not necessarily a precursor to ‘psychopathy’ in adulthood (Hyde & Dotterer, 2022).

Recent findings support the notion of two distinct variants of CU/psychopathic traits with unique etiological pathways and that can be identified using a clustering analytic approach (e.g., latent profile analysis) with measures of CU/psychopathic traits and anxiety and/or trauma as indicators (see review by Craig et al., 2021). Compared to individuals with the *primary variant* whose callousness/unemotionality is linked to genetically-based deficits in emotional arousal and responsivity, individuals with the *secondary variant* appear to have a more emotionally-reactive disposition but have learned to ‘switch off’ their emotions after exposure to abuse, neglect, and violence (Porter, 1996). The ongoing developmental impact of these past experiences of interpersonal trauma helps explain why the secondary variant is associated with relatively more serious and complex mental health problems, including traumatic stress, depression, substance use, and suicidality (Craig et al., 2021). Meta-analytic findings also demonstrate a stronger link between child maltreatment and CU traits at higher levels of anxiety, providing additional support for the distinct CU variants (Todorov et al., 2023).

### Callous-Unemotional/Psychopathic Traits and Alternative Care

Prior reviews of mental health and relational outcomes associated with AC have not discussed CU/psychopathic traits, perhaps due to the limited explicit research on this topic. In addition, given the lack of attention to this subject, it is also likely that there is an unexplored yet emerging evidence base on CU/psychopathic traits in children and young people with experiences of AC. This is because individuals in out-of-home care have been included in studies concerning CU/psychopathic traits and externalizing problems as high-risk samples (e.g., Berg et al., 2013; Smith et al., 2011), though explicit associations between CU/psychopathic traits and AC experiences have not frequently been a key research focus. Overall, this has led to a significant gap in our understanding of developmental pathways and outcomes of CU/psychopathic traits in children exposed to chronic and severe trauma, with important implications for tailored and targeted interventions to support this highly vulnerable population.

**Fig. 1:**
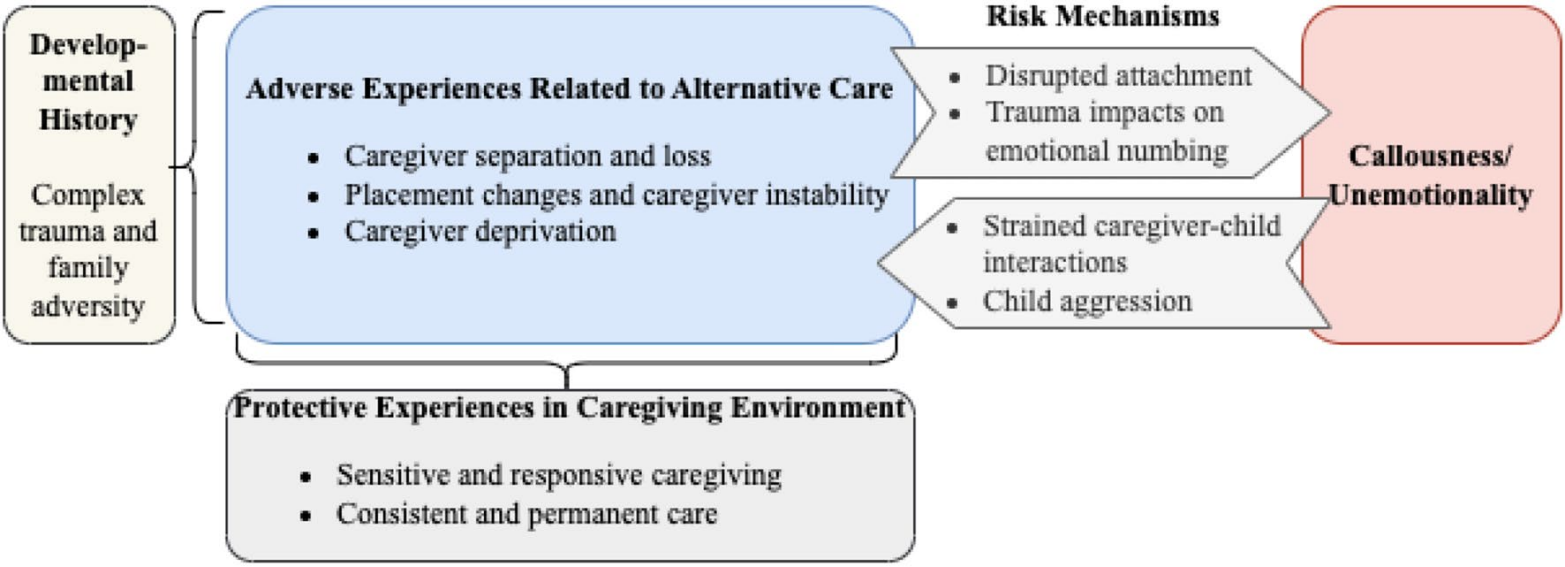
A conceptual relational model linking experiences of alternative care and callousness/unemotionality

There is strong theoretical rationale for investigating CU/psychopathic traits specifically in individuals with experiences of AC. Although there is heterogeneity in developmental timing of and reasons for children entering AC (e.g., maltreatment, parental instability, parental death, poverty; Humphreys, 2019), they all share the profound experience of caregiver loss or separation. Moreover, this grief and trauma is often compounded by severe abuse and neglect and other adversities during development, including ongoing caregiver disruptions (Almas et al., 2020; Briggs-Gowan et al., 2019). The conceptual relational model depicted in Fig. 1 posits that the interpersonal stress and trauma experienced before, on entering, and while living in, AC, may increase risk for CU/psychopathic traits. Moreover, these traits might also impact negative relational experiences in AC and later outcomes. Below, we draw on developmental theories of attachment and trauma, and relevant empirical findings, to help elucidate the nature of key risk and protective processes in the proposed model which provides a framework for the current review.

#### Attachment Disruptions Associated With Alternative Care

Children form attachments with primary caregivers for survival and psychological security. In formulating attachment theory, Bowlby (1973, 1980) observed that young children separated from primary caregivers may eventually become detached (e.g., defensively avoid their caregiver) after initially feeling upset and helpless. Moreover, he suggested that prolonged separation, rejection, and abandonment in the caregiver-child relationship, can lead to anger and rage when children’s desire for connection is not met. In circumstances involving significant caregiver separation, children may struggle to trust in the emotional availability of existing and new caregivers and develop an insecure attachment marked by avoidance or anxious-escalation of their attachment needs (Bowlby, 1973, 1980). Children in AC who have experienced severe trauma or psychosocial deprivation, particularly institutionalized children, are at increased risk for disorganized attachment wherein they lack any reliable strategy for seeking out their caregiver to alleviate distress (Van Ijzendoorn et al., 2011). Disorganized attachment, in turn, may confer risk for deficits in interpersonal behavior and social-emotional functioning, such as lowered attention to and discrimination of facial emotional expressions (e.g., Forslund et al., 2020). Moreover, impoverished care in institutional settings may predispose children to developing attachment disorders wherein they lack attachment behavior (e.g., fail to seek and respond to comforting) and/or indiscriminately approach unfamiliar adults (Zeanah & Gleason, 2015).

Importantly, the quality of caregiving in AC plays a major role in either perpetuating or alleviating children’s attachment-related problems and insecurity. While caregivers’ sensitivity and responsiveness to children’s needs can help children recover from attachment disruptions and buffer against severe stress, significant separations continue for many children living in AC due to caregiver instability. For instance, in the U.S. most children who spend longer than 2 years in foster care experience several placement changes (U.S. Department of Health & Human Services, 2022), and children raised in institutional care often lack consistent and available caregivers. Ongoing attachment disruptions caused by caregiver instability and discontinuity in care can exacerbate attachment insecurity and externalizing and internalizing problems (Almas et al., 2020; Pasalich et al., 2016).

Bowlby (1944) described the enduring effects of early disruptions in attachment relationships and lack of parental care on shaping ‘affectionless’ traits (e.g., indifference to others’ emotions) in delinquent youth. In support of this early claim, meta-analytic findings suggest that insecure attachment is positively associated with CU/psychopathic traits (Van der Zouwen et al., 2018), and research using clinical samples of children show links between disorganized attachment and CU traits over and above externalizing problems (e.g., Kohlhoff et al., 2020; Pasalich et al., 2012). Overall, attachment disruptions linked to AC, including caregiver loss and separation and ongoing caregiver instability, may increase attachment insecurity and underpin emotional detachment and empathy deficits that characterize callousness/unemotionality.

There is also promise, however, that nurturing and stable caregiving in “family-based” AC settings may help buffer the negative effects of past relational adversities on child outcomes (Zeanah et al., 2017), perhaps extending to CU/psychopathic traits. Importantly, warm, responsive caregiving has been shown to be a significant environmental factor associated with lower CU traits (e.g., Waller et al., 2018).

#### Adaptation to Trauma and Adversity Surrounding Alternative Care

Alongside attachment disruptions, individuals with experiences of AC are typically exposed to other forms of adversities throughout childhood. Complex trauma involves chronic and repeated exposure to multiple types of maltreatment (e.g., abuse, neglect, family violence), often within the attachment relationship, and encapsulates the experience of many individuals who have lived in AC (Greeson et al., 2011). For instance, maltreatment perpetrated by a parent is a common precipitant of an out-of-home care placement, which itself is an attachment-related trauma for many children, and sadly, some children are re-traumatized when they receive pathogenic care while in AC and/or after reunifying to birth parents (Hallett et al., 2021). Moreover, children in institutional care may have been exposed to significant caregiver deprivation and neglect (Gunnar and Reid 2019; Van Ijzendoorn et al., 2011).

According to theory regarding developmental trauma (Van der Kolk, 1996), children try to adapt to chronic stress and trauma by developing various coping strategies to help promote mental health. Past findings support Porter’s (1996) idea that some children and youth who have experienced chronic trauma and relational adversities, learn to disconnect or dissociate from their distress and emotions to avoid and lessen their emotional discomfort and pain. As a result, these children might display a mask of callousness and emotional apathy (i.e., CU behaviors) which could be adaptive for regulating overwhelming emotions in the short term (e.g., Bennett & Kerig, 2014; Kerig et al., 2012). In the longer term, however, their callousness/unemotionality may derail the development of healthy relationships and social-emotional functioning. Prior results also suggest that emotional numbing helps account for associations between betrayal trauma (i.e., victimization within a close relationship) and high CU traits in youth (Kerig et al., 2012), providing further evidence for the effects of attachment-related trauma on elevated CU traits. Overall, trauma-informed accounts regarding the emergence of callousness/unemotionality in individuals exposed to chronic and severe interpersonal trauma are particularly relevant to the developmental pathway underlying the secondary variant of CU/psychopathic traits, and shed further light on possible risk processes for these traits in individuals with experiences of AC.

#### Impact of Callous-Unemotional/Psychopathic Traits on Relational Processes in Alternative Care

Although adverse experiences surrounding AC may increase risk for or exacerbate CU/psychopathic traits, it is also likely that there are bidirectional influences wherein these traits potentially disrupt caregiver-child processes and contribute to poorer outcomes in AC. Indeed, past findings demonstrate reciprocal effects between CU/psychopathic traits and parenting experiences. For instance, co-occurring high levels of externalizing problems and CU traits predict increases in parenting distress (Fanti & Munoz Centifanti, 2014). Furthermore, CU traits are linked to child-to-parent aggression (Kuay et al., 2022). Importantly, both externalizing problems and caregiver stress are associated with negative parenting experiences underlying placement breakdowns in out-of-home care (Konijn et al., 2019). As discussed above, placement changes contribute to discontinuity in care and attachment disruptions and may possibly increase callousness/unemotionality. Taken together, these findings support the notion that CU/psychopathic traits and co-occurring externalizing problems may heighten caregivers’ stress and negatively impact caregiver-child interactions, undermining placement stability in AC. In turn, these experiences might increase risk for negative child outcomes beyond AC. Thus, to sharpen the targets, content, and timing of intervention efforts, it is vital that we gain insight into psychosocial correlates of CU/psychopathic traits in individuals with experiences of AC.

### Current Review

Using the conceptual model described above as a framework, this study aimed to conduct a systematic scoping review of the existing knowledge base on CU/psychopathic traits in individuals with experiences of AC. We chose the methodology of a scoping review as the literature lacks an explicit focus on understanding CU/psychopathic traits in the context of AC, and the body of past research pertaining to this topic is heterogenous regarding sample characteristics, measures, and research design. Moreover, there is a need to conduct a broader review to map the existing knowledge base and identify research gaps to inform future directions. Consistent with our proposed conceptual model, we were also interested in understanding both risk and protective factors for CU/psychopathic traits in the context of AC experiences, as many children in AC show age-appropriate levels of empathy and prosocial behavior, despite their relational adversities (Van Ijzendoorn et al., 2011). The following research questions guided the review and narrative synthesis:

Q1: What are the average levels of CU/psychopathic traits in individuals with AC experiences?
Q2: Are experiences of AC associated with levels of CU/psychopathic traits?
Q3: What are the psychosocial correlates of CU/psychopathic traits in individuals with AC experiences?
Q4: What interventions may reduce or prevent CU/psychopathic traits in individuals with AC experiences?

## Method

Methodology was informed by published guidelines relevant to scoping reviews (e.g., Arksey & O’Malley, 2005; Peters et al., 2015) and adhered to reporting criteria outlined in the PRISMA-ScR checklist developed by Tricco et al. (2018). An a priori protocol was developed and registered with the Open Science Framework on 21 March 2021 (https://osf.io/6ng4v/).

### Search Strategy and Article Selection

Prior to initiating a formal, systematic search process, a small sample (*k* = 7) of key studies relevant to the review were located either through prior author knowledge or preliminary searches. As we later refined the search strategy, the success or failure of a given search strategy to retrieve these studies was used as a brief method of evaluation; the failure to retrieve certain studies would indicate insufficient breadth of search terms. As such, our search strategy was refined until it was able to retrieve all of these key studies.

Nine databases including Scopus, Ovid, PsycArticles, PsycINFO, MEDLINE, Cochrane, PubMed, ProQuest, and Web of Science; were systematically searched for articles up to November 2022. For each database, our search strategy targeted studies that simultaneously satisfied two criteria: (a) the study’s title, abstract, or keywords included at least one term relating to out-of-home care or institutional care, and (b) the study’s full text included at least one term relating to CU or psychopathic traits. The search terms used across the databases are included in Appendix A (Online Resource 1).

**Fig. 2:**
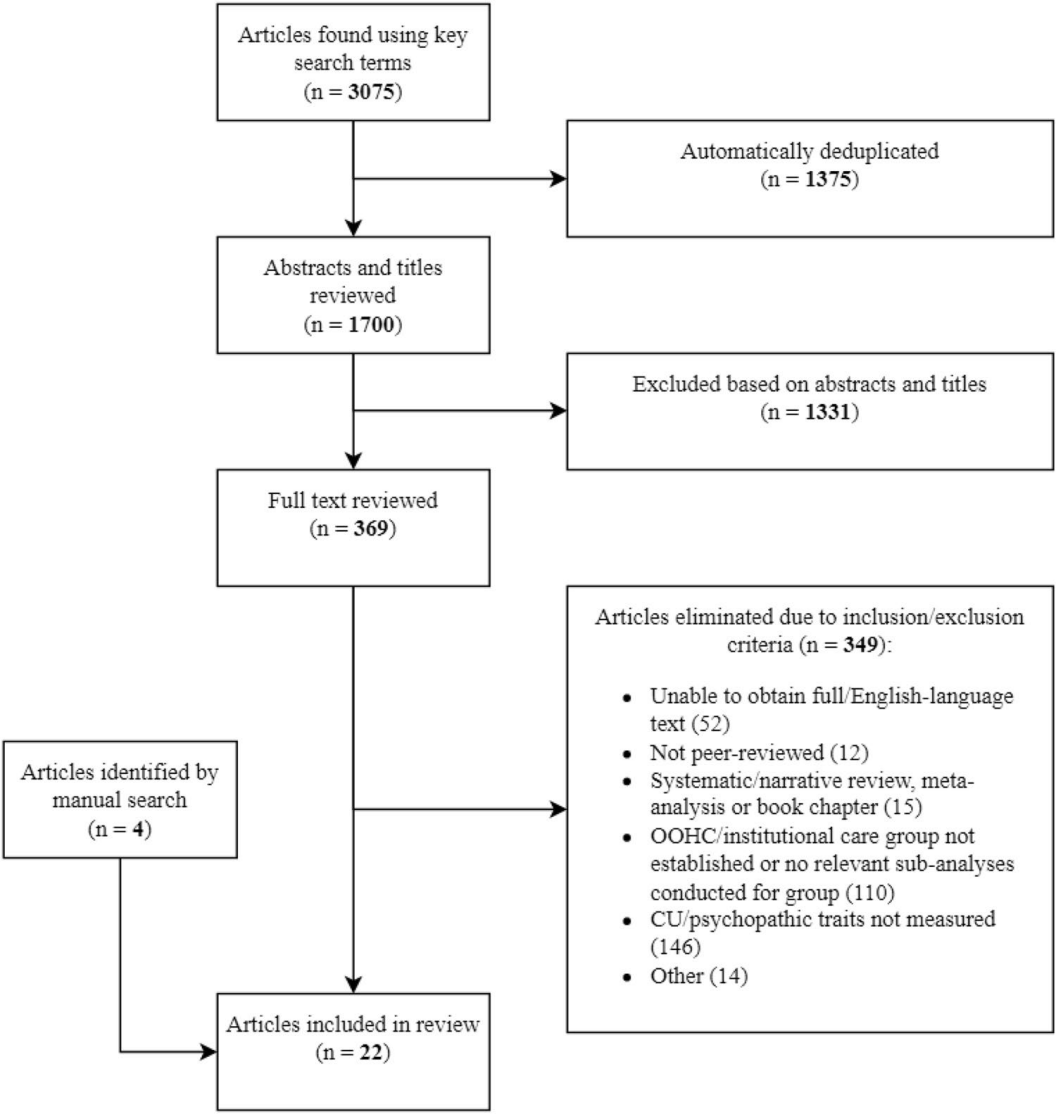
Scoping review search and selection process

Figure 2 provides a detailed description of the study selection process. Selection of studies to be included in the review was conducted across two stages, using the online screening tool, Covidence. Firstly, titles and abstracts of each study obtained from the earlier search were screened by two independent reviewers—once by the primary reviewer (B.A.) and once by a second reviewer. Discrepancies, where the two reviewers did not initially agree on a study’s inclusion/exclusion, were resolved by discussion between the primary reviewer and one of the secondary reviewers. If the conflict could not be resolved by discussion, the study’s lead author (D.P.) was consulted to make a final decision. Resolution of discrepancies was conducted intermittently throughout this stage of screening in order to prevent drift between reviewers and maintain clarity on inclusion criteria.

Secondly, studies retained from the title/abstract stage were then examined in-full (where possible; some studies could not be obtained in-full or in English)—again, once by the primary reviewer and once by any of the secondary reviewers, with discrepancies resolved in the same manner as in the title/abstract stage.

Across both stages of screening, inclusion criteria were as follows: at least one specifiable group of participants experienced AC (for Q’s 1, 3, and 4 only) or a proportion of participants experienced AC (for Q2 only); CU/psychopathic traits were quantitatively measured. Exclusion criteria were: experience of AC could not be established; relevant sub-analyses not conducted for AC group (for Q’s 1 and 3 only); majority of participants in the AC group were adopted from birth; record not peer-reviewed; record not English-language; record is a narrative review, systematic review, meta-analysis or book chapter. The reference lists of studies that met these criteria, thereby passing through both stages of screening, were additionally manually searched for potential eligible records to be included in the review.

### Data Extraction and Organization

As with the screening process, data extraction was also conducted twice independently—once by the primary reviewer and once by one of the other reviewers. The process was initially piloted using only the first three included studies. This provided an early opportunity to revisit the research questions and ensure that our data extraction was best designed to capture relevant information. The data extraction process was further revised throughout as discussions arose in light of specific studies. The following information was extracted (among other fields): sample characteristics, aspects of study design, nature and length of experiences of AC, measures used (including for CU/psychopathic traits), and key findings. Where important data were unclear, the authors of relevant studies were contacted to clarify.

In line with recommendations by Peters et al. (2015), an analysis of study quality was not conducted as our scoping review was intended to provide a comprehensive survey of current empirical knowledge relating to its research questions, regardless of study quality. Furthermore, as many studies containing relevant data were not specifically designed to address our review questions, the overall quality of the study would be unlikely to accurately reflect the quality of the specific data relevant to our review.

Synthesis of data was conducted and reported by research question. In cases where multiple studies presented data regarding the same sample, each study could be included for synthesis if they provided unique information about the sample (e.g., unique variables/measures or analyses), or if the data were collected at a unique timepoint (e.g., in childhood versus young adult). Furthermore, as the primary focus of the review was on callousness/unemotionality as reflected by CU traits and the affective dimension of psychopathy, it was decided that, where possible, dimensions of psychopathic traits that more narrowly focused on this construct (based on face validity) would be prioritized for synthesis over total scores on broader measures of psychopathic traits.

## Results

### Study Characteristics

Of the 3075 records retrieved, 22 studies, published between 2004 and 2022, were included in the review (see Fig. 2). We identified four ‘clusters’ of studies using the same sample. Five studies used the English and Romanian Adoptee (ERA) sample and two studies used the Bucharest Early Intervention Project (BEIP) sample. Both the ERA and BEIP samples included children who were abandoned by their parents and experienced early psychosocial deprivation while in institutional care in Romania. Moreover, three studies used an out-of-home care sample from Missouri (U.S.), and two used a foster care sample from Georgia (U.S.). Overall, studies included samples of children, adolescents, or young adults with experiences of institutional care (23%), residential care (9%), foster/kinship care (50%) or a mixture of AC placements (18%). Of the 12 (55%) studies that reported on race/ethnicity in their samples, participants were predominantly white (*k* = 6), Black (*k* = 5) or ‘Spanish’ (*k* = 1).

Eighty-six percent of included studies reported on a specific scale reflecting callousness/unemotionality in their CU/psychopathic traits measure; the remaining 14% reported on total scores for psychopathic traits. CU/psychopathic traits were most commonly assessed using the Inventory of Callous-Unemotional Traits (ICU; Frick, 2004) (55%), with other studies employing the Psychopathic Personality Inventory-Short Form (PPI-SF; Lilienfeld & Hess, 2001), The Psychopathy Checklist-Revised (PCL-R; Hare et al., 1990), The Psychopathy Checklist-Youth Version (PCL-YV; Forth et al., 2003), Antisocial Process Screening Device-CU scale (APSD-CU; Frick & Hare, 2001), Child and Adolescent Behavior Inventory-Limited Prosocial Emotions subscale (CABI-LPE; Burns et al., 2015), and diagnostic parent interview. Detailed characteristics relating to the studies’ samples, methodologies, and key findings are presented in Appendix B (Online Resource 1). As most included studies contained data relevant to multiple review questions, results from single studies relating to different review questions are reported separately.

### Q1: Levels of CU/Psychopathic Traits in Individuals With AC Experiences

Nine records with unique data were obtained in relation to levels of CU/psychopathic traits in individuals with experiences in AC (Q1). Two of these (belonging to the ERA project) used overlapping samples; however, as the data were collected at two separate timepoints (adolescent and young adult), both studies were retained. Accordingly, eight unique samples were represented from Romania (*k* = 2), U.S. (*k* = 2), Spain (*k* = 2), Canada (*k* = 1), and Israel (*k* = 1).

The ICU was administered in six studies: both parent- and youth self-report (*k* = 2), parent-report only (*k* = 2), youth self-report only (*k* = 1), and teacher-report only (*k* = 1). For administration of the ICU regarding children and adolescents, Kemp et al. (2021) suggested cut-off scores of 24 (self-report), 21 (parent-report), and 35 (teacher-report) to reflect elevated CU traits in the community. These cut-offs were exceeded in three of the five relevant studies to administer the ICU regarding children or adolescents. Specifically, the U.S. sample of adolescents in foster care exceeded both parent- and self-report cut-offs (Berg et al., 2013). Furthermore, in the BEIP and ERA samples, Romanian children who had been exposed to severely-depriving institutionalization exceeded the parent-report cut-off (Humphreys et al., 2015; Kumsta et al., 2012), although the *self*-report cut-off was not exceeded in Kumsta et al. (2012). Two studies in which the ICU was administered regarding children and adolescents found that these cut-offs were not exceeded (Levy et al., 2015; Maneiro et al., 2019). Levy et al.’s (2015) sample of maltreated boys in government housing did not meet the teacher-report ICU cut-off, while Maneiro et al.’s (2019) sample of adolescents in residential care reported ICU levels below the self-report cut-off. However, these two samples still reported mean scores considerably higher than the means obtained in Kemp et al.’s (2021) community sample.

A single study administered the ICU to young adults (Kennedy et al., 2016), using the same ERA sample that was measured in childhood by Kumsta et al. (2012). While Kennedy et al. (2016) did not directly report the overall mean score for those in the sample who had experienced more than 6 months in institutions, we estimated that these participants displayed a mean score of 29.4 on the parent-report ICU, based on the weighted average of two constituent subgroups. We are unaware of established cut-offs for the parent-report version of the ICU in adult populations.

The remaining three studies employed the PPI-SF, CABI-LPE (parent-report), and the PCL-R to measure CU/psychopathic traits. Vaughn et al. (2008) administered the PPI-SF to assess psychopathic traits in a sample of adolescents ageing out of the child welfare system, though eleven items that did not sufficiently load on to their factors were deleted. Their sample’s mean PPI-SF total score of 104.6 would correspond to a total score of 130.2 on the full 56-item measure (when rescaled by number of items; i.e., multiplied by 56/45)—which is approximately half a *SD* greater than the mean total score observed in a community sample of *N* = 1219 U.S. undergraduate students (*M* = 124.04, *SD* = 14.88; Adams et al., 2020).

Navarro-Soria et al. (2020) used the CABI-LPE to measure CU traits, while also including a large comparison sample of children (*N* = 1776), matched in age and country of residence to their foster care sample, that has been used elsewhere for psychometric validation of the CABI (Burns et al., 2021). The foster care sample’s mean score of 2.61 (*SD* = 1.52) on the LPE subscale places above the 80^th^ percentile for boys (*M*[*SD*] = 1.51[1.25]) and above the 85^th^ percentile for girls (*M*[SD] = 1.24[1.18]) in the comparison sample, according to the distribution reported by Burns et al. (2021).

Finally, Forouzan and Nicholls (2015) used the PCL-R to measure psychopathic traits in young women with experiences in foster care. Their foster sample’s mean PCL-R score of 22.08 is slightly below the cut-off of 25 suggested for research purposes; however, 41.46% of their participants scored above this cut-off, and 8.54% scored above 30 (the cut-off score used for forensic purposes; Hare, 1991).

Overall, findings from the nine studies included for Q1 consistently indicate elevated rates of CU/psychopathic traits in individuals with AC experiences.

### Q2: Associations Between Experiences of AC and Levels of CU/Psychopathic Traits

While related to Q1, Q2 was concerned with study designs that investigated the association between AC experiences and CU/psychopathic traits, regardless of whether mean levels were reported. This included comparison group, longitudinal, and retrospective study designs that provide novel information beyond Q1. Eight records were included, with samples from Romania (*k* = 2), Germany (*k* = 2), Finland (*k* = 1), U.K. (*k* = 1), Spain (*k* = 1), and Canada (*k* = 1). Six of the eight relevant studies (Campbell et al., 2004; Humphreys et al., 2015; Joseph et al., 2014; Lindberg et al., 2009; Navarro-Soria et al., 2020; Schutte et al., 2022) examined the association between past or current experiences of AC (coded categorically) and CU/psychopathic traits. Meanwhile, three studies (Krischer & Sevicke, 2008; Kumsta et al., 2012; Navarro-Soria et al., 2020) provided data concerning associations between amount of time or number of placements in AC and levels of CU/psychopathic traits. Notably, Navarro-Soria et al. (2020) reported results from both analytical approaches.

Of the six studies in the first category (i.e., AC experience coded *categorically*), Lindberg et al. (2009) found that among adolescent homicidal offenders, those scoring high ($\ge$ 26) vs. low (< 26) on the PCL-R were more likely to have experienced an institutional or foster care placement in childhood. Similarly, controlling for demographic and criminal history characteristics, Campbell et al. (2004) found that a history of out-of-home care was associated with elevated scores on the PCL-YV. Furthermore, in a sample of adolescents living in either foster or birth families, Joseph et al. (2014) reported an association between foster care group and higher APSD-CU scores, while controlling for age, sex, IQ, maternal education, and single-parent household status. Schutte et al. (2022) also found that young children in foster care had higher ICU scores than their peers in birth families. Navarro-Soria et al. (2020) found a similar pattern of results across different analyses, showing higher CU traits (measured by the CABI-LPE) in children in foster care compared to non-foster/comparison children (controlling for sex and age), though children who had *longer* stays in foster care did not significantly differ in CU traits compared to comparison children when father reports were included for the latter group. Finally, Humphreys et al. (2015) found that Romanian children with a history of institutional care displayed elevated CU traits relative to those raised by birth parents. However, history of institutionalization did not predict CU traits when they were coded dichotomously using an ICU cut-off of 2 *SD*s above the sample mean.

Of the three studies belonging to the second category (i.e., AC experience coded *continuously*), Kumsta et al. (2012) and Navarro-Soria et al. (2020) did not find a significant association between time spent in AC (institutional or foster care) and CU traits measured by the ICU and CABI-LPE, respectively. Moreover, in a sample of incarcerated youth, Krischer and Sevicke (2008) did not find a significant association between number of foster homes and the PCL-YV affective factor when controlling for prior maltreatment and parental antisocial/criminal behavior. However, for girls, number of foster homes was uniquely associated with PCL-YV total score.

Overall, findings from the eight studies relevant to Q2 provide support for an association between higher levels of CU/psychopathic traits and experiences of AC; but not with amount of time in AC. Although the latter finding is based on only two studies, it could be due to study limitations concerning unmeasured individual differences in proximal factors, such as the quality of care children experienced in AC (e.g., see Barone et al., 2016).

### Q3: Psychosocial Correlates of CU/Psychopathic Traits in Individuals With AC Experiences

Sixteen records were identified in relation to Q3. Of these, five were part of the ERA project, two from the BEIP, and three from a research project based in Missouri (U.S.). As these groups each shared a common sample, there were only nine *unique* samples represented in response to Q3, deriving from the U.S. (*k* = 3), Romania (*k* = 2), U.K. (*k* = 1), Canada (*k* = 1), Spain (*k* = 1), and Israel (*k* = 1).

The ICU was again the most frequently used measure of CU/psychopathic traits (*k* = 10), followed by factors of the PPI-SF (*k* = 3; namely, ‘carefree-unemotional’ and ‘coldheartedness/carefree-nonplanfulness’ factors), the APSD-CU subscale (*k* = 1), PCL-R (*k* = 1), and a diagnostic parent interview assessing CU symptoms (*k* = 1).

#### Externalizing Problems

Eight studies examined relations between CU traits and diagnostic categories of externalizing problems and symptoms, with most indicating significant positive associations. Specifically, links between CU traits and oppositional defiant disorder (ODD) were examined in two studies (Humphreys et al., 2015; Kumsta et al., 2012), wherein parent-report ICU scores—coded dimensionally or categorically (i.e., using a cut-off)—evinced a significant positive association with ODD (symptoms or diagnosis). However, youth self-report ICU scores were not significantly associated with ODD diagnosis in youth with experiences in severely depriving Romanian orphanages (Kumsta et al., 2012).

Three studies using the ERA sample examined associations between CU traits—measured by the ICU—and attention-deficit hyperactivity disorder (ADHD) symptoms at their project’s adolescent and young-adult follow-ups. Results showed an association between high CU traits (using a cut-off at age 15) and higher rates of ADHD diagnoses (Kumsta et al., 2012). Similarly, among the adoptees who were exposed to 6 months or more of severe institutionalization, those with vs. without an ADHD diagnosis showed significantly higher CU traits at age 23 (Kennedy et al., 2016). However, Sonuga-Barke et al. (2010) found a positive but non-significant association between CU traits and a measure of ‘inattention/overactivity’ at age 15.

Two studies examined the relationship between CU traits—measured by the ICU—and conduct disorder (CD). Using the BEIP sample, Humphreys et al. (2015) found a significant positive association between parent-report CU traits and CD symptoms and diagnosis at age 12. On the contrary, Kumsta et al. (2012) found that neither parent-report nor youth self-report CU traits were significantly associated with CD diagnosis at age 15 in the overall ERA sample, controlling for IQ and ADHD scores.

Three studies assessed relations between CU/psychopathic traits and specific forms of externalizing problems, and generally reported positive associations. Specifically, Berg et al. (2013) demonstrated that caregiver-report aggressive behavior and rule-breaking were significantly associated with caregiver-report ICU scores, but not youth self-reports. In the overall ERA sample, Kumsta et al. (2012) found that alcohol and tobacco misuse was not significantly different between high- and low-CU trait groups. Furthermore, Levy et al. (2015) found that teacher-report ICU scores were significantly associated with conduct problems, aggression, and prosocial behavior (inversely), although not with salivary oxytocin levels. However, a post-hoc logistic regression analysis suggested that youth with elevated conduct problems and low oxytocin levels were more likely to show high CU traits, though this was limited by a small sample.

Although not exclusively examining associations between CU traits and externalizing problems, an additional two studies (Maneiro et al., 2019; Wade et al., 2021) included CU traits as an indicator in latent profile analysis and found that high CU traits—alongside other externalizing psychopathology and risk factors—contributed to characterizing the highest risk/morbidity class.

#### Internalizing Problems

Three studies with samples of mid-to-late adolescents examined relations between CU/psychopathic traits and various internalizing problems, with two of these reporting significant associations. First, in correlational analyses, Berg et al. (2013) unexpectedly found a pattern of *positive* associations between self-report and caregiver-report ICU scores and measures of psychological distress and other internalizing issues (i.e., anxiety, depression, emotion dysregulation, loneliness, [less] hope), even when controlling for externalizing problems. Further, when examined together in multiple regression, only loneliness and (less) hope were significantly associated with ICU self-report. Second, Kumsta et al. (2012) did not report any significant differences between high- and low-scoring groups on the ICU on rates of anxiety and depression diagnoses in the overall ERA sample. Finally, Smith et al. (2011) found that coldheartedness/carefree-nonplanfulness was significantly positively associated with perceived stress, but not depression symptoms.

#### Attachment-Related Problems

Four records examined relations between CU/psychopathic traits and insecure or problematic attachment and found consistent support for this link. Using a gold standard attachment interview, Joseph et al. (2014) found that adolescents in foster care with insecure vs. secure attachment to their foster caregiver had higher scores on caregiver-report APSD-CU subscale (i.e., greater CU symptoms). In a clinical sample of 20 children with various AC experiences and attachment problems, Mayes et al. (2017) reported that all children (*n* = 15) who had been diagnosed with comorbid attachment disorders (reactive attachment disorder and disinhibited social engagement disorder [DSED]) displayed significant CU traits specified by the DSM-5; whereas, none of the children (*n* = 5) with ‘DSED only’ displayed CU traits. Furthermore, using the ERA sample, Sonuga-Barke et al. (2010) found that disinhibited attachment was significantly positively associated with parent-report ICU scores for 15-year-old adoptees exposed to 6 or more months in a Romanian institution. Similarly, Kennedy et al. (2017) found that Romanian adoptees (*M* age = 23 years) who were exposed to more than 6 months of institutional deprivation *and* showed DSED behaviors in adulthood (e.g., overfamiliarity, poor social boundaries), displayed elevated CU traits relative to those who were deprived at least 6 months but did not show DSED behaviors as well as those who experienced low deprivation (0–6 months).

#### Cognitive Impairments

Two studies using the ERA sample examined associations between CU traits—measured by the ICU—at age 15 and cognitive impairments, finding limited support for this link. Kumsta et al. (2012) found that Romanian adoptees with high CU traits (i.e., above 80th percentile of sample) on average displayed significantly lower IQ levels than those with low CU traits. By contrast, among adoptees who were exposed to at least 6 months of deprivation, Sonuga-Barke et al. (2010) reported that parent-report CU traits were not significantly associated with cognitive impairment, although they were significantly linked with quasi-autism (i.e., pertaining to social-cognitive deficits).

#### Deprivation-Specific Psychological Patterns

Two ERA studies investigated CU traits—measured by the ICU—at age 15 in relation to deprivation-specific psychological patterns (DSPs) (i.e., inattention/overactivity, disinhibited attachment, quasi-autism, and cognitive impairment). Sonuga-Barke et al. (2010) found that, among adoptees exposed to at least 6 months of deprivation, those also diagnosed with a DSP displayed significantly higher parent-report CU traits compared either with participants who had experienced 6 months of deprivation but not developed a DSP, or those who had experienced less than 6 months of deprivation. However, no significant group differences were obtained using *self*-report CU traits. Kumsta et al. (2010) expanded on these findings by examining whether the callous or uncaring dimensions of parent-report CU traits could indirectly account for the relationship between length of institutional exposure and DSP diagnosis. In both cases, no significant indirect effect was found.

#### Pseudo-Prospective Studies

Three studies with older adolescent and young adult samples included pseudo-prospective study designs to examine various psychosocial correlates of CU/psychopathic traits in individuals with AC experiences, and generally demonstrated higher levels of developmental risk factors linked to these traits. Among the three records were two studies that used the same sample of U.S. adolescents ageing out of the child welfare system (Smith et al., 2011; Vaughn et al., 2008), but examined different factors of the PPI-SF.

Firstly, Smith et al. (2011) found that scores on the *coldheartedness/carefree-nonplanfulness* factor at age 19 were associated with the prior (age 17) diagnosis of generalized anxiety disorder and inversely with antisocial personality disorder. It was also inversely associated with being employed or in college by age 19, and positively associated with deviant peers at age 18 and having been arrested for delinquent conduct in the prior 2 years (i.e., age 17–19). However, there was no significant association between coldheartedness/carefree-nonplanfulness and prior diagnosis of post-traumatic stress disorder, substance abuse or history of child maltreatment (all measured at age 17). Secondly, Vaughn et al. (2008) also found that *carefree-unemotionality* at age 19 was positively associated with arrest history over the previous 2 years, with this association remaining significant when controlling for demographic factors, child maltreatment history, deviant peer affiliation, and various other risk factors. By contrast, controlling for the same covariates, carefree-unemotionality significantly decreased the likelihood of having assaulted somebody with a weapon over the past year and was inversely associated with antisocial personality disorder at age 17. Moreover, carefree-unemotionality was not significantly related to having illegally made money or having sold drugs over the past year. Thus, in both of these studies, the CU-related dimensions of psychopathy appear to be linked to moderate delinquent behaviors (e.g., substance use), as opposed to more severe criminal conduct (e.g., drug-dealing or assault with a weapon).

The remaining study used the PCL-R to measure psychopathic traits in a sample of Canadian women (*M* age = 19.6) with a history of foster care (Forouzan & Nicholls, 2015). High vs. low levels of psychopathic traits in late adolescence/young adulthood were examined in relation to 331 psychosocial variables measured across different periods of childhood and adolescence. Results generally showed that, compared with women low on psychopathic traits, those with high psychopathic traits had elevated risk factors at the level of the individual (e.g., early externalizing, internalizing, and cognitive problems, and suicidality; anger and violence to initial foster care placement; criminal behavior in late adolescence), family (e.g., absent or abusive fathers), and school (e.g., problematic teacher-student relationships). Although higher likelihood of paternal abuse was linked to women high on psychopathic traits, in multiple regression, interestingly, exposure to parental neglect was a robust predictor of women with low psychopathic traits.

### Q4: Interventions for CU/Psychopathic Traits in Individuals With AC Experiences

Two randomized controlled trials (RCTs) contained data pertinent to Q4. Firstly, using the BEIP sample, Humphreys et al. (2015) examined intervention effects of high-quality foster care, wherein institutionalized children were placed (at *M* age = 22 months) with Romanian caregivers who received specialized training and support, against a ‘care-as-usual’ group who remained in institutions. At age 12, the two groups did not significantly differ on parent-report ICU using continuous scores; however, for dichotomous ICU scores, children receiving ‘care-as-usual’ were 7.20 times more likely to score above the cut-off than children receiving foster care intervention. Furthermore, the overall intervention effects were stronger for boys than girls, and caregiver responsiveness to distress (but not caregiver warmth) in boys at 42 months significantly mediated the intervention effects on CU traits at age 12.

Secondly, Reddy et al. (2013) tested the effects of cognitively-based compassion training delivered to adolescents (*M* = 14.7 years) in foster care. The 6-week group-based intervention included mindfulness and cognitive behavior therapy components to develop youths’ empathy and acceptance of others. No significant differences in self-report ICU scores (or any other psychosocial measure) were found between the intervention and waitlist control groups. A data collection error meant that data for parent-report ICU at post-treatment were not available.

## Discussion

This paper provides the first conceptual model and comprehensive review of callousness/unemotionality in individuals who have experienced AC. The samples in the included studies consisted of children and young people with experiences of out-of-home care and/or institutional care. We found consistent evidence that, on average, levels of CU/psychopathic traits appear to be elevated in individuals with AC experiences, compared to published norms in community samples and clinical cut-offs. Most studies we reviewed also showed an association between past or current experiences of AC and CU/psychopathic traits, though the causal role of AC in this relationship is difficult to determine as, understandably, studies lacked robust longitudinal designs with baseline measures of these traits prior to entry into AC. As delineated in our conceptual model, regarding non-institutional AC, it is likely that there are bidirectional influences between the quality and stability of caregiving in out-of-home care and callousness/unemotionality.

These findings concerning research Q1 and 2 build on the established literature linking AC to increased rates of various mental health and relational problems (e.g., Engler et al., 2022; Gunnar & Reid, 2019), by suggesting an additional risk for callousness/unemotionality. Importantly, however, most studies included in our review did not control for other dimensions of psychopathology, particularly externalizing problems. Accordingly, it is also possible that externalizing problems may be confounding the associations involving CU traits reported in these studies. Furthermore, it is difficult to understand the specific impacts of AC versus other life adversities on CU/psychopathic traits, given that many children experience maltreatment and other traumatic events prior to entering AC (Greeson et al., 2011; Humphreys, 2019). However, prior findings suggest that caregiver separation and loss—key risk experiences connected to AC—negatively impacts children beyond the effects of violence and other traumatic events (Briggs-Gowan et al., 2019). Arguably, children in AC are a unique population and AC provides a highly influential developmental context for children and should be explicitly recognized and studied in relation to callousness/unemotionality.

Regarding research Q3, we identified a heterogenous group of studies providing data on a range of psychosocial correlates of CU/psychopathic traits in children and young people who have lived in AC. The general pattern of findings demonstrates consistent associations between CU/psychopathic traits and externalizing and internalizing problems. This association with a range of psychopathology, including both hostility and psychological distress, is congruous with the complex mental health problems characterizing the secondary variant of CU/psychopathic traits, which is thought to include an etiology of interpersonal trauma (Craig et al., 2021). Interestingly, studies included in our review reported that, among young people with foster care histories, elevated CU/psychopathic traits were associated with greater anger to initial foster care placement and moderate delinquent behaviors (e.g., substance use), but not serious criminal behavior and antisocial personality disorder (Forouzan & Nicholls, 2015; Smith et al., 2011; Vaughn et al., 2008). Although these findings require replication, they are consistent with the emotionally-reactive disposition and use of maladaptive forms of stress coping in individuals with the secondary variant of CU/psychopathic traits. We speculate that emotional numbing may be a significant coping mechanism for trauma in individuals with AC experiences and could underpin the onset of secondary CU/psychopathic traits in this population.

There was also consistent evidence for an association between CU/psychopathic traits and attachment-related problems in individuals with AC experiences, including insecure attachment with foster caregivers, comorbid attachment disorders, and disinhibited attachment (Joseph et al., 2014; Kennedy et al., 2017; Mayes et al., 2017; Sonuga-Barke et al., 2010). This is in line with the hypothesis in our model that disrupted attachment may be a key mechanism linking adverse experiences related to AC (e.g., caregiver separation and loss, caregiver instability) to CU/psychopathic traits. Findings from a study in our review concerning young women with foster care histories, also point to associations between psychopathic traits and broader risk factors in family-of-origin, including absent or abusive fathers, and interestingly, an inverse relationship with parental neglect (Forouzan & Nicholls, 2015).

In the included studies of Romanian adoptees with a history of institutional deprivation, CU traits were found to be associated with a broad profile of behavioral and cognitive impairments, including quasi-autism (e.g., Kumsta et al., 2012; Sonuga-Barke et al., 2010). Although the specificity of these relations is less clear, it appears that CU traits are highly comorbid with multiple psychological difficulties in populations exposed to early psychosocial deprivation, perhaps reflecting the chronic developmental impact and severity of these unique AC experiences.

Finally, in addressing Q4, we were only able to locate two published interventions. Only the RCT of a foster parent intervention reported benefits for preventing CU traits, and at least in boys, this was accounted for by intervention-induced improvements in caregiver responsiveness to child distress (Humphreys et al., 2015). Importantly, this finding provides experimental support for responsive caregiving as a potential protective factor for CU traits in AC settings. More generally, we were unable to locate any additional studies providing evidence for protective factors for callousness/unemotionality in this population.

### Study Strengths and Limitations

Our study utilized best practice in scoping review methodology and a broad, inclusive literature search to map and critically analyze the existing evidence base on CU/psychopathic traits in individuals with AC experiences. We also integrated a novel conceptual framework to frame the research questions and interpretation of the findings.

Although it is possible that our database search did not identify all the available literature on the subject, the most significant limitations of this review pertain to the limitations of the research designs, analyses, and methodology of the included studies. Most importantly, given the lack of attention on the topic in prior research, many of the studies did not explicitly examine research questions congruous with the ones explored in our review. This has implications for the strength of the findings presented here, including that important covariates or independent variables, such as externalizing problems and early life adversities prior to AC, were typically not examined. These variables are very relevant to understanding the specificity of associations between AC experiences and CU/psychopathic traits, and correlates of these traits in this population. Furthermore, as the majority of data in this review are from cross-sectional or pseudo-prospective studies, causality is difficult to determine. The large heterogeneity in samples across studies also restricts how the findings can be applied to specific AC settings (e.g., foster care, institutional care). Finally, as this is the first review of the literature on callousness/unemotionality in this population, we included studies that measured this construct narrowly via CU traits or more broadly via total scores for psychopathic traits. To enhance comparison among studies, future research on this topic should consider analyzing specific factors (i.e., traits) when psychopathy scales are administered.

### Suggestions for Future Research

This scoping review has revealed several important gaps in the existing evidence base which provide a basis for future research directions. First, to shed light on the specificity and nature of associations between experiences of AC and callousness/unemotionality, future studies should employ longitudinal designs, examine bidirectional effects, and control for potential covariates and other interrelated variables (e.g., externalizing problems, early life adversities prior to entering AC). For example, investigating the bidirectional effects of quality of caregiving and placement stability in out-of-home care and callousness/unemotionality, while controlling for externalizing problems, would help answer questions such as whether CU traits predict increased risk for placement breakdowns in out-of-home care, and vice-versa. Second, research is needed on relational and trauma-coping mechanisms (e.g., attachment disruptions, emotional numbing) potentially accounting for associations between AC and callousness/unemotionality, as well as risk and protective factors predicting distinct developmental trajectories of callousness/unemotionality in children living in AC. Additionally, it would be important to understand whether these mechanisms and risk and protective factors differ in populations with experiences of institutional versus non-institutional AC.

A third future research direction involves intervention trials. Results from the abovementioned lines of research would help guide the selection of existing, and the development of novel, psychosocial interventions that may be well-suited for preventing and reducing callousness/unemotionality in children with experiences of AC. Fourth, future research should factor in broader levels of influences in children’s lives, by examining moderating effects of contextual or system-level factors on the association between AC and callousness/unemotionality. For instance, in out-of-home care, policies favoring kinship care over foster care placements may strengthen mental health and family relationships (Hassall et al., 2021; Winokur et al., 2014), which in turn, could reduce risk for callousness/unemotionality. Moreover, given the known link between out-of-home care and higher likelihood of involvement in the juvenile justice system (e.g., Cutuli et al., 2016), it is possible that callousness/unemotionality may interact with AC experiences to increase risk for this adverse outcome. Results from these moderation studies would have potential to contribute to both practice and policy in child welfare settings.

Finally, to counterbalance the dominant research focus on psychopathology in AC, the field would benefit from a program of strengths-based research examining factors associated with prosocial behavior and empathy, and potential hidden strengths of stress-resistance from emotional numbing, in children living in AC. Although the current review focuses on callousness/unemotionality in AC, many children with experiences of AC demonstrate age-appropriate levels of empathy and prosocial behavior (Van Ijzendoorn et al., 2011); however, there is very little research on protective factors associated with these adaptive interpersonal outcomes. There is significant value for clinical practice in identifying intrapersonal strengths that may co-exist with features of callousness/unemotionality and protective factors that may help buffer risk for the emergence of these features.

### Implications for Clinical Practice

The findings from our review have several important implications for clinical practice in psychotherapy and child welfare settings. Given the consistent evidence for elevated rates of CU/psychopathic traits in children and young people with AC experiences, it is important to appropriately assess these traits or behaviors in clinical practice. Moreover, from the perspective of our conceptual model, it is equally important to adopt a trauma-informed approach to assessing and treating callousness/unemotionality in this population. This involves understanding the child or young person’s unique history of trauma and adversity and how it has contributed to their profile of strengths and difficulties in social-emotional, behavioral, and relational functioning. Applying a trauma-sensitive lens may also reduce potential stigma linked to callousness/unemotionality in AC and encourage a therapeutic response to children’s underlying distress, emotional pain, and relational mistrust; rather than isolated treatment of their symptoms of callousness/unemotionality.

There is also a need for tailored interventions to address comorbid CU/psychopathic traits and externalizing and internalizing psychopathology in children with AC experiences. Interventions informed by attachment and trauma theories appear particularly relevant, given these children’s attachment-related problems found in this review and their prevalence of trauma. Our review identified the promise of training and supporting foster parents in high-quality caregiving for preventing CU traits in individuals with a history of institutional deprivation (Humphreys et al., 2015). Another example of a suitable intervention is the Connect program (Moretti, 2020)—a trauma- and attachment-informed parent intervention targeting caregiver responsiveness that has proven to be particularly effective for reducing externalizing problems in at-risk children high on CU traits (Pasalich et al., 2022). It has also been adapted for foster and kinship caregivers in out-of-home care (Moretti et al., 2020; Pasalich et al., 2021). Targeting externalizing problems is a priority in AC as they are exacerbated by CU/psychopathic traits and are known to predict placement breakdowns in out-of-home care. In addition to promoting the quality of caregiving, there should also be an emphasis on improving the stability of caregiver-child relationships in AC, as ongoing and consistent relationships with caregivers are known to promote healthy social-emotional and relational outcomes in children impacted by trauma and loss (Zeanah et al., 2017).

## Conclusion

In sum, the findings of this systematic scoping review suggest that on average, levels of CU/psychopathic traits are elevated in children and young people with AC experiences, and that these traits may be associated with AC. In this population, we also found consistent associations between CU/psychopathic traits and externalizing and internalizing psychopathology and attachment-related problems, as well as links with other risk factors (e.g., family adversity) and psychological difficulties in development. This pattern of findings provides evidence for the importance of examining callousness/unemotionality in individuals with experiences of AC, in both research and practice. As argued from the standpoint of our conceptual model, future research is needed to provide insight into relational and trauma-based risk mechanisms, as this will help inform tailored and targeted psychosocial interventions for callousness/unemotionality in children and young people who have lived in AC.

## Supplementary Information

Below is the link to the electronic supplementary material.

Supplementary file1 (DOCX 36 KB)

## Footnotes

1 The terms institutional care and residential care are often used interchangeably in the literature (e.g., Roche, 2019); however, this review distinguishes between these two forms of AC settings given their differences in caregiving environment.
