# Supplementary material for: Childhood Experiences of Alternative Care and Callousness/Unemotionality: A Conceptual Model, Scoping Review, and Research Agenda
Source: Clin Child Fam Psychol Rev. 2023 Jul 12;26(3):789–804. doi: 10.1007/s10567-023-00445-4 (PMC10465668; doi:10.1007/s10567-023-00445-4)
Supplement: Supplementary file 1 — Supplementary file1 (DOCX 36 KB) [file 10567_2023_445_MOESM1_ESM.docx]

Online Resource 1

**Childhood Experiences of Alternative Care and Callousness/Unemotionality: A Conceptual Model, Scoping Review, and Research Agenda**

Dave S. Pasalich^1^, Benjamin Aquilina^1^, Alison Hassall^1^, Natalie Goulter^2^, Nakiya Xyrakis^1^ & Anderson Khoo^1^

^1^School of Medicine and Psychology, Australian National University

^2^School of Psychology, Newcastle University, UK

Correspondence concerning this article should be addressed to Dave S. Pasalich, School of Medicine and Psychology, Building 39, Science Rd Canberra ACT 2601, Australia. Email: [dave.pasalich@anu.edu.au](mailto:dave.pasalich@anu.edu.au)

**Appendix A**

Search Terms for Systematic Scoping Review

Table A.1 lists the search strings used in the systematic search for this review. Studies retained from the search for screening were required to meet two criteria. Specifically, they (a) included one or more terms from String 1 in their title, abstract, or keywords, and (b) included one or more terms from String 2 anywhere in the full text (including title, abstract, keywords, etc.).

Table A.1

*Search Strings Used to Retrieve Potential Studies for Screening*

| String | |
| --- | --- |
| 1 | 2 |
| "foster care*" or "foster child*" or "foster parent*" or "abandoned child*" or "family-type home" or "out-of-home care" or "kinship care*" or "foster child*" or "foster parent*" or "kinship parent*" or "resource parent*" or "looked after" or "custodial care" or "alternative care" or "home-based" or "institutionali*" or "institutional care" or "child care institution" or "orphan*" or "children's home" or "residential care" or "group care" or "group home*" | "callous*" or "psychopathy" or "psychopath" or "psychopathic" or "sociopath*" or "unemotional" or "meanness" or "proactive aggression" or "fearless*" or "limited prosocial" |

**Appendix B**

Study Characteristics and Relevant Findings of Included Articles (N = 22)

| **Author (Year)** | **Sample** | **Country of Sample** | **Type of Alternative Care (AC)** | **Experience of AC Is Retrospect-ive (Y/N)** | **Measure(s) of CU/Psychopathic Traits** | **Design** | **Review Question(s) Addressed** | **Findings** |
| --- | --- | --- | --- | --- | --- | --- | --- | --- |
| Berg et al. (2013) | 70 adolescents aged 13-17; 44.3% female; 74.3% African American. | U.S. | Foster care | N | ICU (self and caregiver-report) | Cross-sectional | 1, 3 | (1) ICU-self: *M*(*SD*) = 24.06(8.57). ICU-parent: *M*(*SD*) = 30.92(10.16).  (3) Positive associations between ICU self-report and caregiver-report scores and externalizing (eg, aggression, rule-breaking) and internalizing problems. Regarding the latter, when ICU self-report scores were regressed on anxiety, depression, loneliness, emotional dysregulation, and hope; only loneliness and (less) hope were significant factors. |
| Campbell et al. (2004) | 226 adolescents aged 12-19 in youth detention centres; 17% female; 83% White. | Canada | Relative/kinship care (23.6%); foster care placement (25.9%) | Y | 18-item version of PCL-YV | Cross-sectional | 2 | (2) History of out-of-home care was associated with higher PCL-YV scores. This association remained significant after controlling for demographic and criminal history characteristics. |
| Forouzan & Nicholls (2015) | 82 women aged 18-25 removed from family during childhood and raised in Youth Centres; 100% White. | Canada | Foster care | Y | PCL-R (cut-off of 25) | Cross-sectional | 1, 3 | (1) PCL-R: *M*(*SD*) = 22.08(6.95).  (3) High compared with low levels of adult PCL-R scores were associated with multiple psychosocial risk factors across childhood and adolescence, including: early childhood psychological and behavioral problems, paternal absence and abuse, parental neglect (inversely), school issues, and antisocial and criminal behavior in late adolescence. |
| Humphreys et al. (2015) | BEIP sample, 145 Romanian children; 51.0% female; data from follow-up at age 12.  Ever-institutionalized group randomly allocated to: high-quality foster care (*n*=48) or to ‘care as usual’ (continued institutionalisation) (*n*=47). Control group (*n* = 50) was never institutionalised. | Romania | Institutional care, with subset entering foster care at age 5-31 months | Y | ICU (parent-report), cutoff 2 SDs above mean | Longitudinal (Q1, 2 & 4); Cross-sectional (Q3) | 1, 2, 3, 4 | (1) Foster care: boys *M*(*SD*) = 20.38(9.39); girls *M*(*SD*) = 22.42(8.11). Care-as-usual: boys *M*(*SD*) = 27.74(11.14); girls *M*(*SD*) = 21.86(10.41).  (2) Controlling for sex, ever-institutionalised group scored higher on CU traits (dimensionally) than never-institutionalised group, but not more likely to score above ICU cutoff.  (3) Within ever-institutionalised group: associations between CU traits and ODD and CD symptoms. Using ICU cutoff also indicated positive association between high CU traits and CD diagnosis.  (4) CU traits in foster care intervention group not significantly different from care-as-usual, except when using ICU cutoff – care-as-usual children 7.20 times more likely to have high CU traits. In boys only, care-as-usual significantly higher on CU traits (dimensional and cut-off scores) than foster care. In boys only, foster care intervention effect on CU traits mediated by caregiver responsiveness to child distress (at 42 months), but not caregiver warmth. |
| Joseph et al. (2014) | 112 adolescents aged 10-17.  62 in foster care; 46.8% female; 54.8% Caucasian.  50 in comparison group; 48.0% female; 68.0% White. | U.K. | Foster care | N | APSD-CU subscale (caregiver and teacher report) | Cross-sectional | 2, 3 | (2) Foster care group scored higher on CU traits, while controlling for age, sex, IQ, maternal education, and single-parent household status.  (3) Foster youth with insecure vs. secure attachment to foster mother displayed higher (caregiver-reported) CU traits. |
| Kennedy et al. (2016) | ERA sample, 217 participants; 50.5% female; data from follow-up at age 23.  165 adoptees with history of institutional deprivation in Romania.  52 never-exposed adoptees from U.K. | Romania and U.K. | Institutional care | Y | ICU (parent-report) | Longitudinal (Q1); Cross-sectional (Q3) | 1, 3 | (1) *M*=29.39 (weighted average of sub-group means) for ≥ 6 months institutionalization.  (3) Participants split into three groups: ADHD+ (6+ months’ institutionalization combined with ADHD), ADHD- (6+ months’ institutionalization but no ADHD), and LoDep (0-6 months’ institutionalization). ADHD+ significantly higher on CU traits than either ADHD- or LoDep groups. No significant differences on CU traits between adult-onset vs. persistent ADHD. |
| Kennedy et al. (2017) | ERA sample, 164 participants; 50.0% female; data from follow-up at age 23. 122 adoptees with history of institutional deprivation in Romania.  42 never-exposed adoptees from U.K. | Romania and U.K. | Institutional care | Y | ICU (parent-report) | Longitudinal (Q1); Cross-sectional (Q3) | 1, 3 | (3) Participants split into three groups: DSE+ (6+ months’ institutionalization combined with disinhibited social engagement [DSE]), DSE- (6+ months’ institutionalisation but no DSE), and Low Deprivation (LoDep) (0-6 months’ institutionalization). At age 23, DSE+ scored significantly higher on CU traits than either DSE- or LoDep. DSE- not significantly different from LoDep. |
| Krischer & Sevicke (2008) | 185 adolescents aged 14-19 in juvenile justice facilities; 48.1% female; 86% White. | Germany | Foster care | Y | PCL-YV (affective factor) | Cross-sectional | 2 | (2) For boys, girls, and total delinquent sample, number of foster homes was not associated with the PCL-YV affective factor when controlling for prior maltreatment and measures of parental antisocial outcomes. However, for girls only, number of foster homes was uniquely associated with PCL-YV total score. |
| Kumsta et al. (2010) | ERA sample, 129 – 136 participants; 50.0% female; data from follow-up at age 15. Adoptees from Romania, exposed to severe institutionalisation; never-exposed adoptees from U.K. | Romania and U.K. | Institutional care | Y | ICU (self- and parent- report) | Cross-sectional | 3 | (3) Subscales of parent-report ICU not found to account for the following indirect effects pathway: length of institutional exposure $\to$ callous or uncaring CU dimension $\to$ deprivation-specific psychological pattern (DSP; quasi-autism, cognitive impairment, inattention/overactivity, and disinhibited attachment). |
| Kumsta et al. (2012) | ERA sample, 117 – 135 participants; 50.0% female; data from follow-up at age 15. Adoptees from Romania, exposed to severe institutionalisation; never-exposed adoptees from U.K. | Romania and U.K. | Institutional care | Y | ICU (parent and self-report), cut-off 80th percentile | Longitudinal (Q1 & 2); Cross-sectional (Q3) | 1, 2, 3 | (1) Among Romanian group: ICU-parent M(SD) = 26.07(13.2), ICU-self M(SD) = 20.39(7.5).  (2) Time spent in institution not significantly different between high- and low-CU trait groups.  (3) High-CU trait group: lower IQ, higher rates of ADHD diagnosis. High and low CU groups not significantly different on rates of depression, anxiety, and alcohol and tobacco misuse. Controlling for confounds, ICU-self not significantly associated with either ODD or CD diagnoses. ICU-parent associated with ODD but not CD. |
| Levy et al. (2015) | 67 adolescent males aged 15-19. | Israel | Residential care for socially at-risk youth | N | ICU (teacher-report), cut-off 80th %ile | Cross-sectional | 1, 3 | (1) ICU-teacher: M(SD) = 33.14(9.47).  (3) Significant associations between CU traits and: conduct problems, aggression, prosocial behaviour (inverse). No significant association with salivary oxytocin. Non-significant interaction between conduct problems and oxytocin. However, in post-hoc analysis, increased likelihood of high CU traits in individuals with elevated conduct problems and low oxytocin, relative to those low on conduct problems. |
| Lindberg et al. (2009) | 114 male homicidal offenders; 57 adolescents; 57 adults (comparison) | Finland | Institutional care or foster care at any point in childhood | Y | PCL-R, cut-off = 26 | Cross-sectional | 2 | (2) Adolescent boys with high vs. low PCL-R scores were more likely to have experienced an institutional or foster care placement in childhood (58% versus 31%). |
| Maneiro et al. (2019) | 145 adolescents aged 11-19; 40.7% female; 85.5% Spanish. | Spain | Residential care | N | ICU (self-report) | Cross-sectional | 1, 3 | (1) Estimated *M* on ICU based on weighted latent profile analysis classes: 21.62.  (3) Alongside other psychosocial risk-factors for antisocial behavior, CU traits were entered as an indicator for latent profile analysis. Three risk-taking classes were obtained. High-risk class (17%) significantly higher on CU traits than middle-risk (33%) and low-risk (50%) classes, and characterized by highest levels of risk factors (eg, violent attitudes, narcissism). |
| Mayes et al. (2017) | 1848 children aged 4-17 referred to a psychiatric clinic. 20 were removed from biological parents or adopted from orphanages in Russia or China, and diagnosed with RAD and/or DSED; 50.0% female; 70.0% White. Other subsamples (not reported on in this review): 933 children with autism and 895 with ADHD. | U.S. | Foster, adoptive and/or institutional care | N | Diagnostic parent interview combined with Pediatric Behavior Scale to code presence/absence of DSM-5 “Limited Prosocial Emotions” criteria, cutoff 2+ criteria met. | Cross-sectional | 3 | (3) All children (*n* = 15) meeting criteria for reactive attachment disorder (RAD) also had disinhibited social engagement disorder (DSED), and 100% had high CU traits (and 73% had comorbid CD). The children (*n* = 5) with DSED only, did not display CU traits (or CD symptoms). |
| Navarro-Soria et al. (2020) | 1825 children aged 8-13. 49 in foster care; 57.0% female; 51.0% White. 1776 in comparison group recruited from elementary schools; 49.0% female. | Spain | Foster care | N | Child and Adolescent Behavior Inventory-Limited Prosocial Emotions subscale (CABI-LPE) | Cross-sectional | 1, 2 | (1) Foster care group CABI-LPE: M(SD)=2.61(1.52).  (2) Foster care group displayed significantly higher LPE compared to the scores reported by comparison group mothers or fathers, controlling for sex and age. No significant association between number of months in foster care and LPE. Foster children with shorter stays (i.e. below the ‘medium months in care’) had higher LPE than comparisons. Similarly, foster children with longer stays (i.e. above the ‘medium months in care’) had higher LPE than comparisons, but not when father reports of LPE were used for comparison children. |
| Reddy et al. (2013) | 71 adolescents aged 13-17; 56% female; 78.8% African American. | U.S. | Foster care | N | ICU (self- and parent-report) | Longitudinal | 4 | (4) Non-significant effect of cognitively-based compassion training vs. waitlist control on CU traits (self-report). No data available for parent-report CU traits at post-treatment. |
| Schutte et al. (2022) | 234 children aged 2-7. 86 in foster care; 52.0% female. 148 in birth families; 51.0% female. | Germany | Foster care | N | ICU (parent report) | Cross-sectional | 2 | (2) Foster children showed higher CU traits than children in birth families. |
| Shook et al. (2009) | 404 adolescents aging out of child welfare system; 57.0% female; 51.0% African American. Assessments at age 17, 18, and 19. | U.S. | Out-of-home care placements | N | PPI-SF (carefree-unemotional factor) | Longitudinal | 1, 3 | (3) Three latent classes of participants were derived based on level of deviant peer affiliation (DPA). The high-DPA class scored significantly higher on the carefree-unemotional factor than the low-DPA class. |
| Smith et al. (2011) | 1885 adolescents. 404 in out-of-home care; 57.0% female; 51.0% African American. Assessments at age 17 and 19. Other subsamples (not reported on in this review): 723 juvenile delinquents and 758 undergraduate students. | U.S. | Out-of-home care placements | N | PPI-SF (coldheartedness/carefree-nonplanfulness factor) | Cross-sectional | 3 | (3) Among the out-of-home care sample: Significant associations between coldheartedness/carefree-nonplanfulness at age 19 and perceived stress at age 19 and deviant peers at age 18. No significant association with perceived family support at age 19 or depression symptoms. Coldheartedness/carefree-nonplanfulness at age 19 was associated with employed/in college at 19 (inverse), arrested between age 17-19, as well as GAD and ASPD diagnosis (inversely) at age 17. No significant associations with PTSD or substance abuse diagnosis at age 17, or history of child abuse/neglect. |
| Sonuga-Barke et al. (2010) | ERA sample, 129 – 136 participants; 50.0% female; data from follow-up at age 15. Adoptees from Romania, exposed to severe institutionalization; never-exposed adoptees from U.K. | Romania and U.K. | Institutional care | Y | ICU (self- and parent-report) | Cross-sectional | 1, 3 | (3) Among participants exposed to 6+ months of institutionalization, parent-report CU traits at age 15 significantly positively associated with disinhibited attachment and quasi-autism, but not with cognitive impairment or inattention/overactivity. Participants split into three groups: DSP+ (6+ months’ institutionalization combined with deprivation-specific psychological pattern [DSP]), DSP- (6+ months’ institutionalisation but no DSP), and comparison (0-6 months’ institutionalisation). No significant differences between the three groups on self-report ICU. Using parent-report ICU, DSP+ scored higher on CU traits than either the comparison or DSP- groups. |
| Vaughn et al. (2008) | 404 adolescents aging out of child welfare system; 57.0% female; 51.0% African American. Assessments at age 17 and 19. | U.S. | Out-of-home care placements | N | PPI-SF (carefree-unemotional factor) | Cross-sectional | 1, 3 | (1) Carefree-unemotionality: M(SD) = 26.3(8.3). (3) After controlling for demographic factors, employment, family support, substance abuse, childhood trauma, deviant peers, ADHD and neighborhood disorder, carefree unemotionality at age 19 was significantly associated with: number of arrests over past 2 years, likelihood of having assaulted with a weapon over past 1 year (inversely), and likelihood of age 17 ASPD diagnosis (inversely). |
| Wade et al. (2021) | BEIP sample, 165 Romanian children; 51.0% female; data from follow-up at age 12. Ever-institutionalized group randomly allocated to: high-quality foster care (*n* = 56) or to ‘care as usual’ (continued institutionalization) (*n* = 57). Control group (*n* = 52) was never institutionalized | Romania | Institutional care, with subset entering foster care at age 5-31 months | Y | ICU (parent-report) | Cross-sectional | 3 | (3) As revealed through latent profile analysis, high morbidity class (9.4%) at age 12 characterized by relatively high levels of CU traits, externalizing, internalizing, ADHD, and relatively low IQ. 93.3% of youth in high morbidity class had a history of institutionalization |

*Note*. ICU = Inventory of Callous-Unemotional Traits; PCL-YV = Psychopathy Checklist-Youth Version; PCR-R = Psychopathy Checklist-Revised; APSD-CU = Antisocial Process Screening Device-CU scale; CABI-LPE = Child and Adolescent Behavior Inventory-Limited Prosocial Emotions subscale; PPI-SF = Psychopathic Personality Inventory-Short Form; ODD = oppositional defiant disorder; CD = conduct disorder; ADHD = attention-deficit/hyperactivity disorder; GAD = generalized anxiety disorder; ASPD = antisocial personality disorder; PTSD = post-traumatic stress disorder.

**Appendix C**

Summary of Measures Reviewed of CU/Psychopathic Traits

| **Measure(s) of CU/Psychopathic Traits** | **Number of Items** | **Informant in Reviewed Studies** |
| --- | --- | --- |
| Inventory of Callous-Unemotional Traits (ICU; Frick, 2004) | 24-items | Caregiver, teacher, self-report |
| Psychopathic Personality Inventory-Short Form (PPI-SF; Lilienfeld & Hess, 2001) | 45- to 56-items  across studies | Interview/self-report |
| The Psychopathy Checklist-Revised (PCL-R; Hare et al., 1990) | 20-items | Observer (e.g., semi-structured interview; file review) |
| The Psychopathy Checklist-Youth Version (PCL-YV; Forth, Kosson, & Hare, 2003) | 18- to 20-items  across studies | Observer (e.g., semi-structured interview; file review) |
| Antisocial Process Screening Device-CU scale (APSD-CU; Frick & Hare, 2001) | CU subscale: 6-items | Caregiver and teacher-report |
| Child and Adolescent Behavior Inventory-Limited Prosocial Emotions subscale (CABI-LPE; Burns et al., 2015) | LPE subscale: 4 items | Caregiver-report |
| Diagnostic parent interview combined with Pediatric Behavior Scale (Lindgren & Koeppl, 1987) to code presence/absence of DSM-5 “Limited Prosocial Emotions” criteria | Cutoff 2+ criteria met | Parent-interview |

Lindgren, S.D., & Koeppl, G.K. (1987). Assessing child behavior problems in a medical setting: Development of the Pediatric Behavior Scale. In R.J. Prinz (Ed.), *Advances in Behavioral Assessment of Children and Families* (Vol. 3., pp. 57–90). JAI Press.
